# Covid-19 Infection in India: A Comparative Analysis of the Second Wave with the First Wave

**DOI:** 10.3390/pathogens10091222

**Published:** 2021-09-21

**Authors:** Arnab Sarkar, Alok Kumar Chakrabarti, Shanta Dutta

**Affiliations:** Division of Virology, ICMR-National Institute of Cholera Enteric Diseases (ICMR-NICED), P-33, C.I.T. Road, Scheme-XM, Beliaghata, Kolkata 700010, West Bengal, India; arnabsarkar2006@gmail.com (A.S.); drshantadutta@gmail.com (S.D.)

**Keywords:** COVID-19, SARS-CoV-2, first wave, second wave, clades

## Abstract

Coronavirus disease 2019 (COVID-19) is considered as the most dreaded disease that has spread all over the world in the recent past. Despite its outbreak in December 2019–January 2020, a few continents and countries such as India started to experience a significant number of COVID-19-positive cases from March 2020. GISAID clade variation analysis in the period March 2020–February 2021 (period I) and March 2021–first week of April 2021 (period II) showed a rapid variation of SARS-CoV-2 in all continents and India over time. Studying the relationship of patient age or gender with viral clades in these two periods revealed that the population under 10 years of age was the least affected, whereas the 11–60-year-old population was the most affected, irrespective of patient gender and ethnicity. In the first wave, India registered quite a low number of COVID-19-positive cases/million people, but the scenario unexpectedly changed in the second wave, when even over 400,000 confirmed cases/day were reported. Lineage analysis in India showed the emergence of new SARS-CoV-2 variants, i.e., B.1.617.1 and B.1.617.2, during April–May 2021, which might be one of the key reasons for the sudden upsurge of confirmed cases/day. Furthermore, the emergence of the new variants contributed to the shift in infection spread by the G clade of SARS-CoV-2 from 46% in period II to 82.34% by the end of May 2021. Along with the management of the emergence of new variants, few factors viz., lockdown and vaccination were also accountable for controlling the upsurge of new COVID-19 cases throughout the country. Collectively, a comparative analysis of the scenario of the first wave with that of the second wave would suggest policymakers the way to prepare for better management of COVID-19 recurrence or its severity in India and other countries.

## 1. Introduction

SARS-CoV-2, a highly contagious coronavirus, expanded from Wuhan, China to all over the world within few months and by the end of March 2020, COVID-19 spread was considered as the first wave of infection. Isolation, sequencing and phylogenetic analysis confirmed the severe acute respiratory syndrome-coronavirus-2 (SARS-Cov-2) as a novel etiological agent which belongs to the same family of SARS-CoV-1 and sequence-wise close to bat derived Yunnan/RaTG13/2013 coronavirus strain [1]. To date, seven human coronaviruses have been isolated, of which OC43, NL63, HKU1 and 229E [2] cause self-limited mild symptoms, while SARS, MERS and SARS-CoV-2 are responsible for severe respiratory syndromes [3]. Phylogenetic studies showed that the rodent is the primary host for HCoV-43 and HKU1, while bat is the primary host for the remaining five viruses [4]. The primary host is the place where genetic recombination, deletion, insertion, missense mutations, etc. give rise to different variants which may be capable of infecting other hosts such as dogs, camels and humans. In this article, we will focus on SARS-CoV-2, more specifically on the infectivity of SARS-CoV-2 in humans.

Proteomic analysis of the first SARS-CoV-2 isolated in Wuhan revealed the presence of four major viral proteins, i.e., the spike surface glycoprotein (S), the envelope protein (E), the matrix protein (M) and the nucleocapsid protein (N). Moreover, 16 non-structural proteins (NSP1-16) and 8 accessory proteins viz., 3a, 3b, p6, 7a, 7b, 8, 9b, 9c, and 10 were also found in SARS-CoV-2 [5,6]. Structural and biochemical analysis disclosed that the S protein, crucial for virus binding with the host receptor, comprised two subunits- S1 and S2. The S1 subunit (14–684 residues) contains an N-terminal domain and a receptor-binding domain (RBD). The RBD domain of S1 has an affinity for the ACE2 receptor of humans and other species. The junction between S1 and S2 has a polybasic cleavage site (RRAR) and during viral infection, cleavage of this junction is carried out by the host serine proteases TMPRSS2 [7]. 

The S1/S2 cleavage site is the key region for determining virus infectivity and mutations (deletion/missense) in S1/S2 cleavage site generated more transmissible SARS-CoV-2 variants [8]. The SARS-CoV-2 strain isolated in Wuhan, China, continually modifies itself by random mutations, developing new variants with higher transmissibility and severity. To track the genetic diversity of SARS-CoV-2, GISAID has introduced a nomenclature of viral clades. Although at the onset of the pandemic, there were three clades—O, S, and L—soon the situation changed with the appearance of six additional clades, namely, G, GR, GH, GRY, GV, and V [9,10]. Clade G was defined as carrying the D614G mutation in the spike protein; the other clades, which emerged in September 2020 and spread globally, were defined as follows: GH (S: D614G, NS3: Q57H), GR (S: D614G, N: G204R), GV (S: D614G, S: A222V) and GRY (S: H69del, S: V70del, S: Y144del, S: N501Y, S: D614G, N: G204R) [9].

Irrespective of the clade, SARS-CoV-2 viral proteins are capable of activating the secretion of pro-inflammatory cytokines including IL-6 and IL-8 [11] at the onset of infection. At the late stage of infection, uncontrolled secretion of pro-inflammatory cytokines results in the generation of cytokine storm and associated multi organ failure [12]. However, the impact of COVID-19 infection is not restricted to the physical health of the patient but is also evident on patient’s mental health. Patients suffering from chronic autoimmune diseases such as Systemic Lupus Erythematosus and Hashimoto’s disease experience pandemic-related anxiety and depression and are at risk of insomnia [13,14]. The continual evolution in the viral genome has made COVID-19 unmanageable over the time because of its more contagious nature, and the virus has developed the ability to evade neutralizing antibodies, which has contributed to the surge of the fatality rate (CFR).

While the first case of COVID-19 in India was registered in January 2020 [15], it took more than a year to complete the first wave of infection. During the prolonged first wave, India registered a low number of daily confirmed cases/million inhabitants in comparison to many other countries; however, the scenario started changing from March 2021, with the rapid rise of COVID-19-positive cases throughout the country. A comparison of COVID-19 positive cases/day between India and the rest of the world, as represented in the repository ‘ourworldindata’, showed that the first wave in India started in March 2020, achieved a peak in September 2020 with more than 90,000 confirmed cases/day, and gradually decreased in intensity with 10,000 confirmed cases/day in February 2021. Except for few countries including India, most of the other countries/continents witnessed the first wave of COVID-19 before August 2020 while the second wave started appearing in August–September 2020 [16] followed by the third wave in March 2021, which is still ongoing [17].

A comparative analysis of the number of COVID-19-positive patients in India with respect to other continents showed that India was behind or missed one wave that raised from September 2020 to February 2021. In this article, we comprehensively examined the scenario in India and comparatively analyzed the global COVID-19 dataset to describe clade variation, patient age and gender distribution, emergence of new lineages, and the Government’s stringent policies in the first and the second wave of COVID-19.

## 2. Results

### 2.1. Global Distribution of Clade Variations in March 2020–February 2021 (Period I) and March 2021–First Week of April 2021 (Period II) 

**Figure 1:**
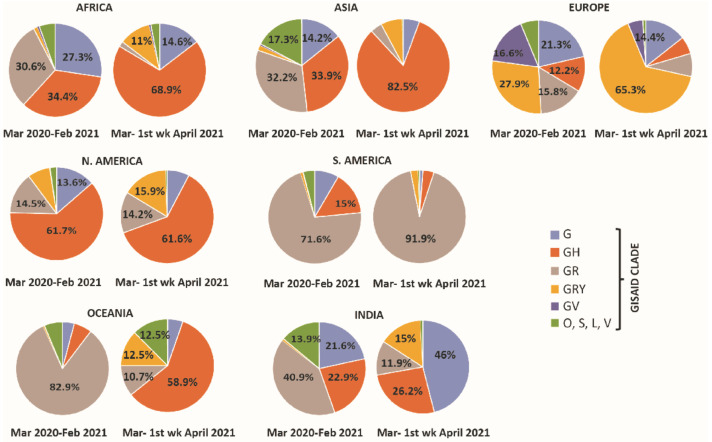
Clade distribution in all continents including India. The clade distribution pattern revealed that over time, the prevalence of the GH clade was higher in Africa, Asia, North America, and Oceania in March 2021–first week of April 2021 (period II) as compared to March 2020–February 2021 (period I). Similarly, in the second period, infectivity of the GRY and GR clades were higher in the European and South American populations respectively. The analysis clearly revealed an increased percentage of the G clade in the second period of infection as compared to the first period in India.

In this study, metadata were downloaded from the GISAID EpiCoV™ Database. We categorized the globally collected COVID-19 patient data into period I and period II, during which India faced the first and second waves of COVID-19infection respectively. Continent-wise analysis in period I (n = 2, 76,049) showed that among all clades, GR was highly prevalent in Oceania (82.8%) and South America (71.5%) and GH diffused in three continents, i.e., North America (61.73%), Africa (34.4%) and Asia (33.9%), whereas, GRY was mostly prevalent in Europe (27.9%). Surprisingly, clade distribution completely transformed over time, as in period II (n = 55,029), the GH clade became the most common clade in Asia (82.5%), Africa (68.9%), North America (61.65%) and Oceania (58.9%). Although the high prevalence of the GH clade in Oceania in period II looks surprising, this is not the case for Asia, North America and Africa, as this clade dominated these continents earlier in period I. On the other hand, the GRY and GR clades were mostly prevalent in Europe (65.3%) and South America (91.9%) in both the periods (Figure 1). Lineage analysis in Oceania revealed the absence of the prevalent D.2 lineage (GR clade) of SARS-CoV-2 in period II, which might be one of the key reasons for the variation in clade distribution. Clade distribution also varied in India with a shift from the highly prevalent GR clade (40.09%) in period I to the G clade (46%) in period II. As clade variation corresponded to the emergence of new variants or the disappearance of prevalent variants, PANGOLIN lineage analysis in these two periods in India revealed that the outbreak was due to the emergence of B.1.617 and B.1.618 SARS-CoV-2 variants, which might be responsible for the variation in clade distribution.

### 2.2. Relationship of Patient Age or Gender with SARS-CoV-2 Clade Variations: A Global Analysis

#### 2.2.1. Patient Age

**Figure 2:**
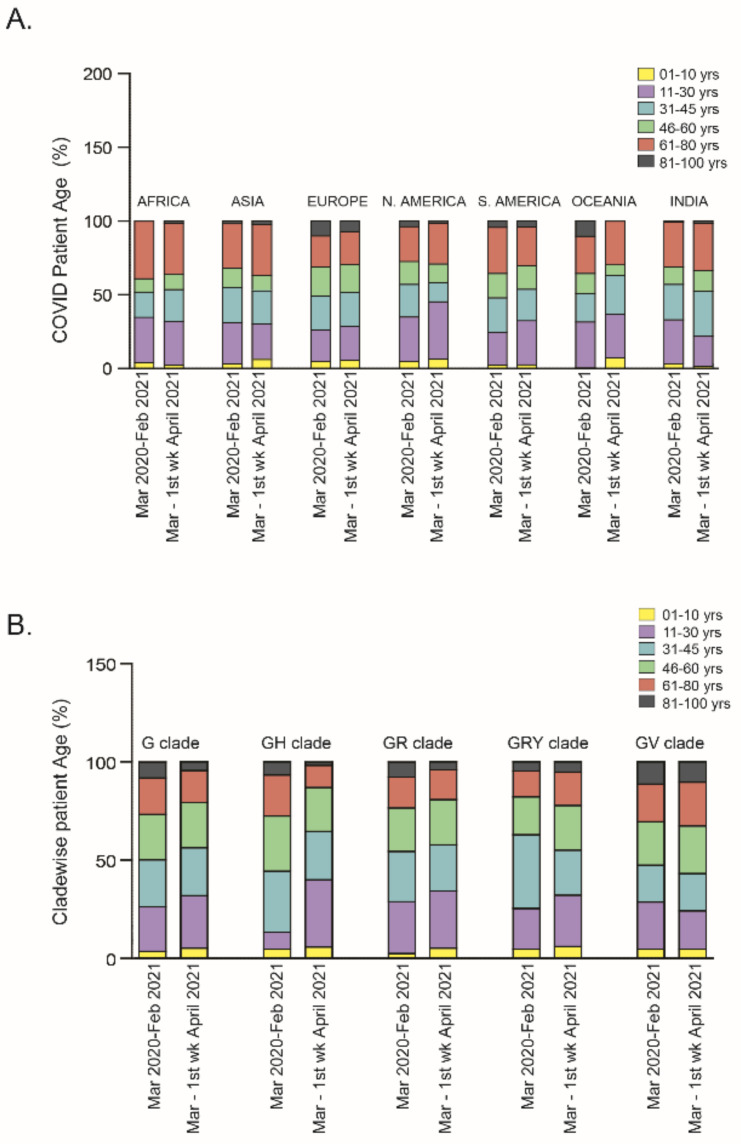
(**A**) Stacked diagram of patient populations with different ages, arranged continent-wise for both period-I (March 2020–February 2021) and period-II (March 2021–first week of April 2021). (**B**) For both periods, the percentage of COVID-19 patients grouped according to their age was plotted in relation to infection with each specific clade (G, GH, GR, GRY and GV).

Patient age is considered as one of the important factors for hospitalization and mortality [18]. Irrespective of gender and ethnicity, older patients appear to be at higher risk of mortality than younger patients. However, in terms of viral transmission, all age groups were equally susceptible to COVID-19 infection [19]. In a recent report, it was claimed that the population under 35 years of age has a high risk of SARS-CoV-2 infection [20]. In the dataset used in the current study, epidemiologically categorized patient data were compared with COVID-19 patient age in periods I and II. The analysis showed that in both periods, three age groups, viz., 11–30 years, 31–45 years and 46–60 years were the most affected followed by the 61–80 years, 81–100 years and 1–10 years age groups. A comparative analysis showed the most affected population was aged 31–45 years in Africa and Asia and 11–30 years in North America and Oceania. However, in Europe, three age groups, i.e., 11–30 years, 31–45 years and 46–60 years appeared to be equally vulnerable in both periods of infection. Surprisingly, in South America, the most affected age group in period I was the 31–45 years (31.23%), though in the following two months it was the 11–30 years (29.73%) (Figure 2A). Similarly, in India the most highly infected patient age groups were the 11–30 years (30.01%) and the 31–45 years (30.48%) in period I, which changed to the 31–45 years (32.03%) and the 46–60 years (30.47%) groups (Figure 2A) in period II of COVID-19. 

To test whether the viral clade has any impact on the age group, clade-wise analysis was performed and it was found that the GH clade (currently the most prevalent SARS-CoV-2 clade) infected a higher percentage of the people of the 11–30 years group, from 8% in period I (odds ratio 0.3, CI 95% 0.29–0.31) to 34.3% in period II (odds ratio 1.41, CI 95% 1.38–1.49) (Figure 2B). In contrast, the percentage of GRY clade-infected patients in the 31–45 years group was reduced from 37.52% in period I (odds ratio 1.25 CI 95% 1.21–1.28) to 22.85% in period II (odds ratio 0.93 CI 95% 0.89–0.97) (Figure 2B). Notably, children under 10 years of age were affected less compared to adults [21] irrespective of ethnicity and clade. In this study, clade-wise and continent-wise analysis also showed that children under 10 years were at low risk of infection in both the periods of COVID-19 spread.

#### 2.2.2. Patient Gender

**Figure 3:**
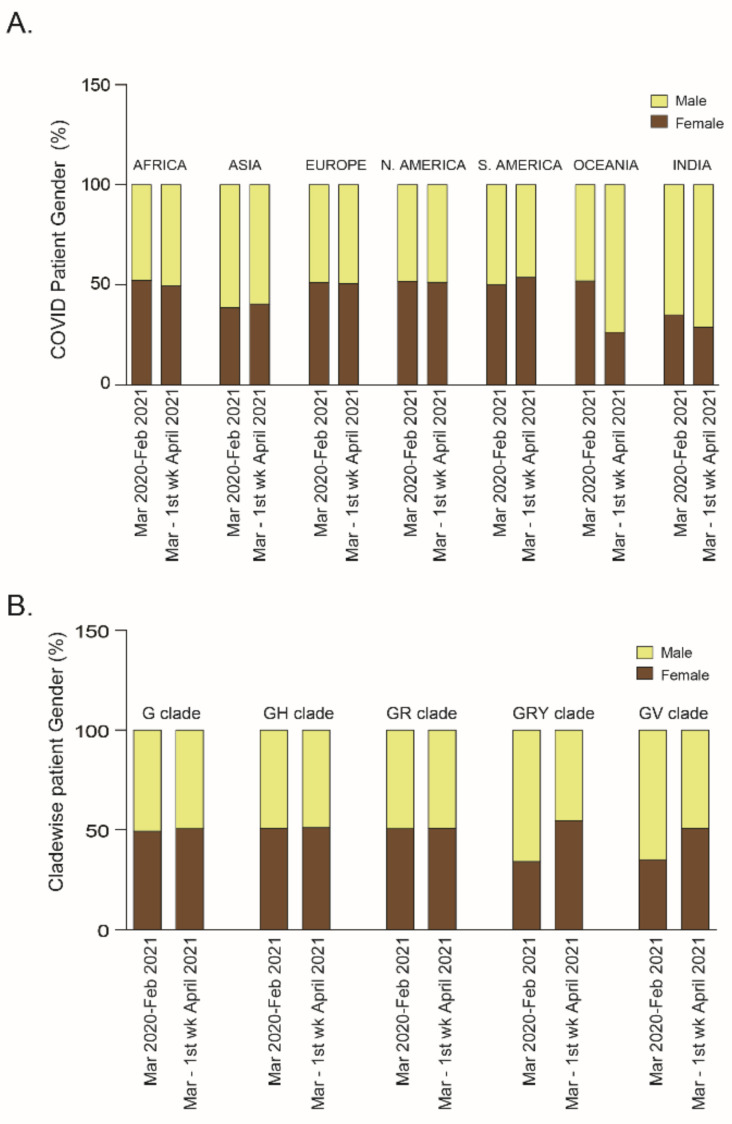
(**A**) Continent-wise and (**B**) clade-wise analysis based on COVID-19 patients’ gender for both period I (March 2020–February 2021) and period II (March 2021–first week of April 2021).

A literature survey revealed that male patients were more strongly affected than females in terms of mortality or disease severity [22,23]. However, with respect to COVID-19 incidence and patient gender, no significant relation has been reported [24,25] so far. To study whether COVID-19 spread was affected by gender, confirmed cases in all continents were arranged depending on patient’s gender in the two periods of this study; no increased susceptibility in relation to gender was found. However, India and the rest of the Asia registered a higher number of male than female patients in both period I (male patients: Asia, 61.4%, India, 64.9%) and period II (male patients: Asia, 59.54%, India, 71.09%). Interestingly, COVID-19 spread in Oceania showed no significant relationship to gender in period I (female patients, 51.9%, male patients, 48.08%) but the percentage of male patients was higher (73.68%) than that of the female patients (26.3%) in period II (Figure 3A). To understand these changes, PANGOLIN lineages were studied and it was found that the new lineage B.1.466.2 infected preferentially the Oceanian male population (n = 19) than the female (n = 1) population but infected equally males and females in the Asian community. Therefore, more information may be required for any further conclusion.

To identify any relationship between clades and COVID-19 patient gender, a clade-wise analysis was performed. A dataset analysis showed no significant relationship between G, GH, and GR with patient gender in both periods. However, the male population was more likely to be susceptible to the GRY (odds ratio 1.83 CI 95% 1.78–1.88) and GV clades (odds ratio 1.71 CI 95% 1.66–1.75) in period I as compared to period II (GRY: odds ratio 0.85 CI 95% 0.82–0.88; GV: odds ratio 1.05 CI 95% 0.94–1.18) (Figure 3B).

### 2.3. Phylogenetic Analysis of the First and Second Waves of COVID-19 Infection in India

**Figure 4:**
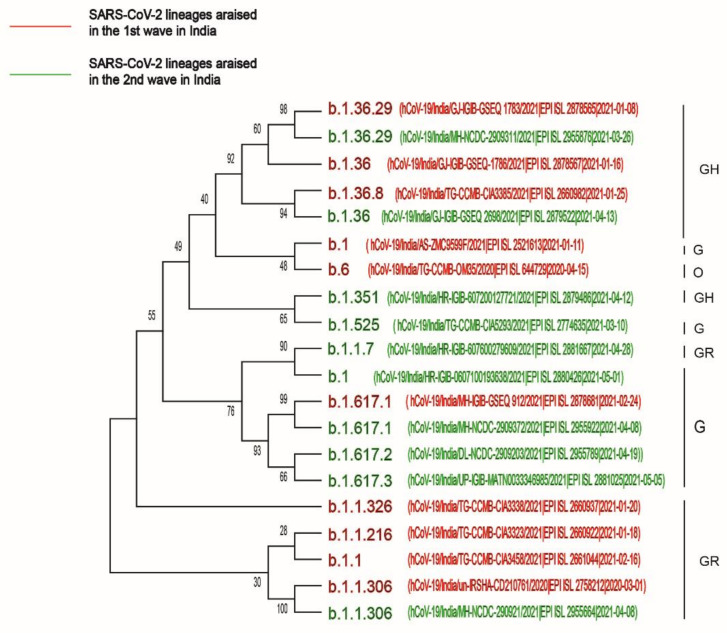
Phylogenetic analysis of 10 highly prevalent SARS-CoV-2 lineages in the two waves of infection in India. Red color designates highly prevalent lineages in the first wave, and green color designates highly prevalent lineages that appeared in the second wave of infection.

To determine the route of evolution of the highly infective SARS-CoV-2 variants, whole-genome sequences of 10 highly prevalent variants in the first wave of infection (B.1.36, B.1.1.306, B.1, B.1.36.29, B.1.1, B.1.1.216, B.1.617.1, B.1.1.326, B.1.36.8, B.6) and 10 highly prevalent variants in the second wave of infection in India (B.1.617.2, B.1.617.1, B.1.17, B.1, B.1.617.3, B.1.36.29, B.1.351, B.1.525, B.1.1.306) were downloaded from the GISAID repository and analyzed by the MEGA-X software. The results showed that the SARS-CoV-2 variants of the GH and GR clades were highly prevalent in the first wave of infection. However, in the second wave of infection, the G clade infected the Indian population at a higher rate as compared to the GH and GR clades. Moreover, the phylogenetic analysis revealed that B.1.617.1, present in both the waves, was the evolutionary ancestor of B.1.617.2 and B.1.617.3 (Figure 4) and might be responsible for the variation in clade distribution in India from the GR clade to the G clade, as shown in Figure 1.

### 2.4. Current Scenario (April 2021–May 2021) in India

**Figure 5:**
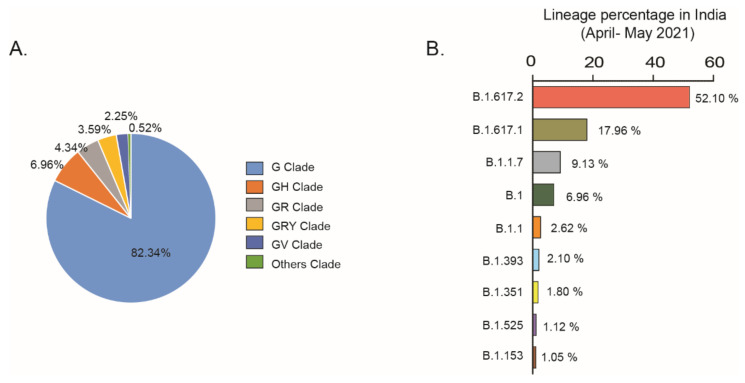
(**A**) COVID CG analysis showed that in the period of April–May 2021, G clade infection increased from 46% to 82.34%; (**B**) lineage analysis shows the percentages of SARS-CoV-2 PANGOLIN lineages in India in the period of April–May 2021.

In India, COVID-19-positive cases increased and touched a peak during the period of April–May 2021. However, in comparison to the rate of infection, the number of deaths/million Indian inhabitants is not alarming. As the emergence of highly propagating SARS-CoV-2 lineages may contribute to the rise of COVID-19 cases, it is essential to study the appearance of new variants and clades in April–May 2021 in India. Analysis using the COVID-19 CoV genetic tool (COVID-19 CG), an open-source tool for visualizing GISAID SARS-CoV-2 data, showed a sudden increase of infections due to the G clade (82.34%) in the period of April–May 2021 (Figure 5A). The spread of the G clade corresponded to the outbreak of the new variant carrying the D614G spike mutation, which is capable of infecting people at a higher transmission rate than other clade variants. Lineage analysis showed that in this period, B.1.617.2 (52%) and B.1.617.1 (17.96%) were the two prevalent SARS-CoV-2 variants in India (Figure 5B).

In Table 1, we listed the nine most prevalent variants in India and studied their mutation status. Among these, the B.1.617.1 and B.1.617.2 variants were most prevalent in India compared to the rest of the World. Analysis based on an available public resource (https://outbreak.info/situation-reports, accessed on 27 May 2021) showed that B.1.617.2 has mutations in ORF1b, S, ORF3a, ORF7a, ORF8, M and N genes, as shown in Table 1. Location-wise analysis showed that the prevalence of the B.1.617.2 lineage in India was 14% as compared to 1% worldwide. Similarly, B.1.617.1 has mutations in ORF1b, S, ORF3a, ORF7a and N genes and this lineage has been highly prevalent in India (19%) in comparison to the World (5%) [26].

### 2.5. Government Stringent Policies

The emergence of new SARS-CoV-2 variants in the second wave of infection may be considered as a predominant reason for the rising of COVID-19 confirmed cases in the Indian community as compared to the first wave. The implementation of Government’s stringent policies was another key factor for COVID-19 case management in both periods I and II. To track the transmission of infectious diseases, a real-time reproduction number (Rt) is calculated depending on the number of cases. Rt > 1 indicates that the infection transmission is progressing exponentially [27]. During the first wave, nearly all countries’ governments calculated the Rt on a daily basis to follow up viral transmission and implement stringent policies. Near all countries except Sweden and Japan, implemented partial or full lockdown on the onset of the COVID-19 outbreak. Lockdown has several positive effects such as (1) it could break the chain of the exponential increase of virus infection by inhibiting the rates of contact between people [28]; (2) provided time to strengthen countries’ medical capacity, such as obtaining test kits and personal protective equipment (PPE) kit, organizing quarantine centers, increasing the number of beds and ventilation equipment etc.; (3) as high dust or air particulates may act as virus carriers [29], a long lockdown markedly reduced pollution as well as viral transmission; (4) could limit viral diffusion, since as reported, high ground ozone levels and low nitrous oxide levels favor viral growth in summer (April–June), a time when India was under full lockdown [30]. 

**Figure 6:**
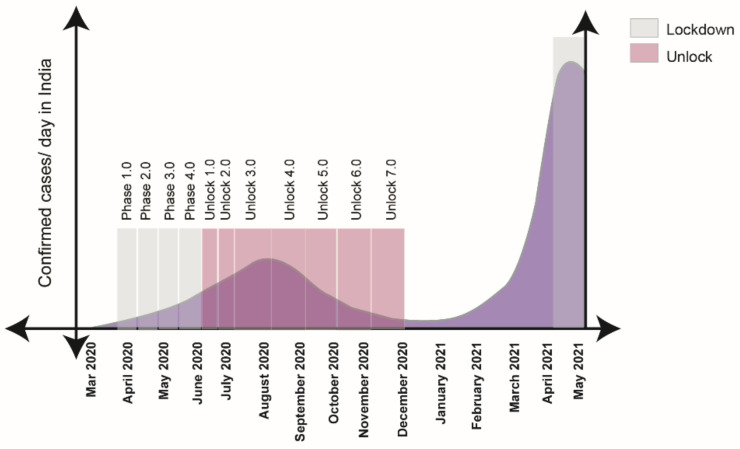
Diagram showing that in the first wave, the nationwide lockdown in India started at the end of March and lasted up to June 2020, followed by unlocking from the end of June 2020 to December 2020. In second wave, the state-wise lockdown has been implemented from April 2021 to the present.

A detailed description of the lockdown and the unlock phases in India is presented in Figure 6. With the onset of the first wave in India, the Government imposed a lockdown all over the country in four phases, i.e., Phase 1: 25 March–14 April 2020, Phase 2: 15 April–3 May 2020, Phase 3: 4 May–17 May 2020, Phase 4: 18 May–31 May 2020. The lockdown was gradually withdrawn in several steps to balance the need to provide safety from the virus and restore the economic growth of the country. Lockdown withdrawal phases were as follows: Unlock 1.0: 30 May–8 June 2020, Unlock 2.0: 1 July–31 July 2020, Unlock 3.0: August 2020, Unlock4.0: September 2020, Unlock 5.0: October 2020, Unlock 6.0: November 2020, Unlock 7.0: December2020. Over time, India started experiencing a low number of CODID-19-positive cases and during February 2021, the number of COVID-19 confirmed cases/day was very low, which indicated that the first wave of COVID-19 was close to an end. However, the unexpected sharp rise of confirmed cases towards the end of March 2021 indicated the initiation of the second wave in India. State-wide lockdown was found beneficial for managing COVID-19 confirmed cases in the second wave of infection as after few weeks of the implementation of the lockdown, the number of confirmed cases/day dropped evidently.

Along with lockdown implementation, early tracing of variants, maintaining strict preventive measures like wearing a face mask, frequent use of sanitizers, maintaining social distance of at least six feet, increasing the number of tests/day, ensuring the availability of test kits, performing timely serosurveys were found helpful to restrict the spread of new variants to other states.

The Government of India started to vaccinate the community in January 2021, which might be a key factor to restrict community transmission and reduce case fatality rate (CFR). The government continually encourages the public to be vaccinated against COVID-19, as experts estimate that if 80–90% of the population is vaccinated or has recovered from COVID-19, herd immunity would be achieved. However, there is always a disagreement as to whether COVID vaccines create herd immunity; in fact, continuous viral transmission and variants generation, as well as waning of vaccine-induced immunity are the three hurdles for achieving the herd immunity threshold [31]. The average numbers of daily vaccinations and average daily full vaccinations are summarized in Table 2. A rapid vaccination is the only way to limit community transmission and restrict COVID-19 recurrence in India.

## 3. Discussion

The goal of this study was to comprehensively analyze the key factors responsible for the sharp rising of confirmed COVID-19 cases in India in the second wave of infection as compared to the first wave. Dataset analysis explored the link between gradual changes in clade distribution due to virus mutations and the emergence of new SARS-CoV-2 variants globally. Continual mutations in spike gene, ORF1a, ORF1b, ORF3a, ORF8, N gene, E gene and M gene generated different clades (G, GH, GR, GRY), leading to the different degrees of virulence across the world. The frequent determination of viral load in patients and associated clades of the circulating viral strains may be crucial to understand the molecular evolution of the virus and make decisions regarding COVID-19 treatment. Our study showed that clade distribution in India varied over time, with a rise of the G clades from 21.6% in period I to 46% in period II and an escalation to 82.34% by the end of May 2021. COVID CG analysis confirmed that during the period of April–May 2021, the rapid emergence of few lineages such as B.1.617.2 (52%) and B.1.617.1 (17.96%) of the G clade might have been responsible for the expansion of the G clade in India. Phylogenetic analysis confirmed that the B.1.617.1 lineage evolutionary generated the B.1.617.2 and B.1.617.3 lineages in the second wave of infection in India. As the G clade was associated with a higher viral load than other clades [32], it can be inferred that the rapid rising of the G clade in India during period II of infection played a significant role in augmenting the rate of COVID-19 infection. Previous studies showed that infection with SARS-CoV-2 clade L/V was strongly associated with the generation of pro-inflammatory cytokines [33], severe COVID-19 lung infection [34] and increased creatinine levels in COVID-19 patients [35]. On the contrary, infection with viral clades carrying the 614G amino acid mutation in the spike protein did not increase patient severity significantly [32]. Therefore, the high number of daily confirmed cases but the low number of deaths /million Indian individuals could be explained by the high distribution of the G clades across India in period II of COVID-19 infection. 

Studying the distribution of the SARS-CoV-2 clades in relation to patient age indicated that all viral clades infected patients irrespective of their age. However, in terms of mortality, G clades viral infection led to mortality in 37.43% of older patients in 58 countries [36]. The presence of comorbid factors like hypertension, cardiovascular disease, deep venous thrombosis and chronic renal disease in older patients may have contributed to the rise of patient mortality due to COVID-19 [37]. Our study showed that patients in the age groups of 11–30 years, 31–45 years and 61–80 years were at high risk of COVID-19 infection in both periods across the world. Moreover, the clade-wise analysis showed that the 11–30-year-old population was at high risk of infection with the GH clade in period II of COVID-19 spread.

A metadata analysis by Hamid et al. concluded that disease severity and mortality in male patients were higher than those in female patients of the same age [38]. One report explained that this gender bias was due to a dwindling T cell activation system in older males compared to older females [39]. Our analysis revealed that except for India and the rest of Asia, the two genders were equally infected in periods I and II of COVID-19 spread across the world. However, the clade-wise analysis showed that in period II, both genders were equally susceptible to infection globally.

Along with the emergence of highly transmissible variants in India, the ‘implementation of lockdown’ factor need to be discussed. In India, the calculated Rt in the first week of March 2020 was 3.2 and further stabilized to 1.09 in the third week of April when India was under lockdown [40]. The Rt went below 1 from September 2020 to February 2021, when the Government of India relieved the lockdown in the country. In April 2021, the Rt in India reached 1.37, and this time, lockdown was necessary to arrest the virus diffusion [41]. State-wise lockdown proved effective in terms of managing daily COVID-19 cases, as imposing lockdown in April–May 2021 lowered the number of COVID-19 confirmed cases/day from 400,000 cases on 8 May 2021 to 127,000 cases on 31 May 2021. 

Along with its strengths, this study has several limitations. The first limitation is that the data derived from the GISAID database do not contain patient clinical history. Several studies showed that diseases like hypertension, diabetes, cardiovascular disease, renal disease may be important comorbid factors influencing patient hospitalization and mortality. Secondly, along with comorbid factors, the presence of influenza A H1N1 and other seasonal coinfections and the type of care provided by different hospital systems need to be considered. Thirdly, the information on the number of recovered and deceased patients in relation to each viral clade was also not available. Fourthly, we did not include asymptomatic or pauci-symptomatic patients in this study. 

Collectively, our results demonstrate that the analysis of viral clade distribution is important to understand changes in COVID-19 clinical manifestations over time. The shifting of highly prevalent viral clades from 1^st^ wave to 2^nd^ wave of COVID-19 infection in India explains the high infection rate per million of individuals. Studies explored that the 31–45-year-old Indian population was infected more extensively in the second wave as compared to the first wave of infection. The implementation of lockdown was one of the most effective strategies to manage viral propagation and helped lower COVID-19 spread in India. In addition, a rapid diagnosis of SARS-CoV-2 variants, strict adherence to preventive measures, prompt diagnosis and treatment, appropriate and rapid launch of vaccination throughout the whole population were equally important to restrict viral transmission in the Indian community.

## 4. Conclusions

The current study indicates the importance of clade variation analysis to understand the evolution of the current COVID-19 pandemic. The analysis presented in this study indicates that the emergence of SARS-CoV-2 clade G was one of the prime reasons for the onset of the second wave and associated increase of COVID-19-positive cases in India, which were >400,000/day in May 2021. Besides the emergence of new SARS-CoV-2 lineages, a comparative analysis also showed the importance of lockdown in preventing the spread of infection in India. Considering the current trend of the pandemic, India is still in the second wave of infection and there is a fear that the third wave will develop. Only time can tell whether the third wave of SARS-CoV-2 infection will hit India, but results from this comparative analysis would definitely encourage to create new and appropriate policies to mitigate COVID-19 severity and manage the second and third waves of the pandemic successfully.

## 5. Materials and Methods

### 5.1. SARS-CoV-2 Metadata Analysis

Deposited COVID-19 information as metadata was downloaded from the GISAID database (https://www.epicov.org, accessed on 21 April 2021). Metadata of COVID-19 included information on date of collection, geographic location, patient gender, patient age, and viral genome clade. Firstly, depending upon the date of collection, the data were categorized into two periods i.e. period I (March 2020–February 2021) and period II (March 2021–first week of April 2021). Only data having proper information viz. patient gender, patient age (1–100 years), and viral isolation from the human host were included in the study. By using ‘filter’ in Microsoft excel, we screened the data and found n = 212,550 out of n = 850,559 and n = 55,029 out of n = 170,776, which were subsequently selected for the current study of period I and period II respectively. All the filtered data used in this study were further assorted depending on the geographic location viz. Africa, Asia, Europe, North America, South America, Oceania and India. The collected data included in the current study were as follows: Africa 8,953 (period I), 164 (period II); Asia 17,724 (period I), 873 (period II); Europe 83,850 (period I), 22,091 (period II); North America 82,969 (period I), 31,429 (period II); South America 8,035 (period I), 419 (period II); Oceania 11,524 (period I), 58 (period II); India 6,438 (period I), 128 (period II). For comparing the changes over these two periods, clades variations i.e. G, GH, GR, GRY, GV and others clades (S, L, V, O), patient age groups (1–10 years, 11–30 years, 31–45 years, 46–60 years, 61–80 years, and 81–100 years) and patient gender (male or female) were further classified in their respective continents or India. 

### 5.2. Case Study of Clades and Lineages in April–May 2021 in India

To analyze the current scenario of COVID-19 spread in India in the period April–May 2021, GISAID Initiative COVID CG (https://covidcg.org, accessed on 27 May 2021) developed by the Broad Institute of MIT and Harvard (USA) was used. Setting of clade or lineages in the period of April–May 2021 in India was used to understand the current scenario of clades and lineages distribution in India [9].

### 5.3. Mutation Status of SARS-CoV-2 Variants in India 

GISAID Initiative ‘Mutation Situation Reports’, developed by the Anderson Lab at Scripps Research (USA), explored the mutation status of nine highly prevalent lineages in India. This data were also used to analyze the prevalence percentages of these SARS-CoV-2 variants in India and worldwide [26].

### 5.4. Vaccination Data Sources

For information regarding vaccination in India, data were collected from the GitHub repository (https://github.com/owid/covid-19-data/blob/master/public/data/vaccinations/country_data/India.csv, accessed on 27 May 2021). The data were divided into groups recording the number of daily vaccinations/month and that of daily fully vaccinations/month. The vaccination data for January–25 May 2021 reflect the current situation of vaccination in India.

### 5.5. Phylogenetic Analysis

Whole genome sequence of SARS-CoV-2 lineages in period I and period II of COVID-19 pandemic in India were downloaded from the GISAID database (www.epicov.org/, accessed on 15 July 2021). The sequences were aligned, and a neighbor-joining phylogeny tree was constructed by using MEGA-X software.

### 5.6. Statistical Analysis

All the data in the figures are presented as percentages. For evaluating the association of a viral clade with population age or patient gender, odds ratios were calculated. An odds ratio > 1 indicated that a clade infection was more likely to be associated with the patient age or gender, whereas an odds ratio < 1 indicated that a clade infection was less likely to be associated with patient age or gender. Confidence of interval (CI) was set at 95%. All calculations were done by using https://select-statistics.co.uk/calculators/confidence-interval-calculator-odds-ratio/software, accessed on 31 May 2021. 

## Figures and Tables

**Table 1:** Mutations of emerged variants of SARS-CoV-2 in April–May in India.

Lineage	Clade	ORF1a	ORF1b	S	ORF3a	ORF6	ORF7a	ORF8	M	E	N	Location Percentage
B.1.617.2	G	-----	P314L, G662S, P1000L	T19R, del157/158, L452R, T478K, D614G, P681R, D950N	S26L	-----	V82A T12OI	Del119/120	I82T	-----	D63G, R203M, D377Y	India (14%), Worldwide (1%)
B.1.617.1	G	-----	T1567I, T3646A, P314L, M1352I, K2310R	L452R, E484Q, D614G, P681R, Q1071H	S26L	-----	V82A	-----	-----	-----	R203M, D377Y	India (19%), Worldwide (0.5%)
B.1.1.7	GRY	T1001I, A17O8D, I2230T, del3675/ 3677	P314L	Del69/70, del144/145, N501Y, A570D, D614G, P681H, T716I, S982A, D1118H	-----	-----	-----	Q27, R25I, Y73C	-----	-----	D3L, R203K, G204R, S235F	India (10%), Worldwide (42%)
B.1	G/GH	-----	P314L	D614G	-----	-----	-----	-----	-----	-----	-----	India (11%), Worldwide (5%)
B.1.1	GR	-----	P314L	D614G	-----	-----	-----	-----	-----	-----	R203K, G204R,	India (7%), Worldwide (3%)
B.1.393	unknown	-----	P314L	D614G	-----	-----	-----	-----	-----	-----	-----	India (1%), Worldwide (0.5%)
B.1.351	GH	T265I, K1655N, K3353R, del3675/3677	P314L	D80A, D215G, del241/243, K417, E484K,N501Y, D614G, A701V	Q57H, S171L	-----	-----	-----	-----	P71L	T205I	India (2%), Worldwide (1%)
B.1.525	G	T2007I, del 3675/3677	P314L	Q52R, A67V, del69/70, del144/145, E484K, D614G, Q677H,F888L	-----	Del2/3	-----	-----	I82T	L21F	T205I, del3, A12G	India (1%), Worldwide (0.5%)
B.1.153	G	-----	-----	D614G	-----	-----	-----	-----	-----	-----	-----	India (1%), Worldwide (0.5%)

Data access to https://outbreak.info, accessed on 23 May 2021.

**Table 2:** COVID-19 vaccination in India (data accessed on 27 May 2021).

Month, 2021	Average Vaccination/Day	Average Full Vaccination/Day
January	2,50,589	0
February	2,88,794	87,721
March	14,17,358	2,21,887
April	23,19,791	5,76,215
Up to 25 May	11,25,377	6,14,969

Data accessed to https://github.com, accessed on 27 May 2021.

## Data Availability

GISAID EpiCoV™ Database was used for analyzing the metadata for further exploration of relevant data. We would like to thank the GISAID Initiative and are grateful to all data contributors, i.e., the Authors, the originating laboratories responsible for obtaining the specimens, and the submitting laboratories for generating the genetic sequence and metadata and sharing them via the GISAID Initiative, on which this research is based [26]. We used the data available in the github repository (github.com/owid/covid-19, accessed on 27 May 2021) for the collection of vaccination data in India.

## References

[1] Biswas A., Bhattacharjee U., Chakrabarti A.K., Tewari D.N., Banu H., Dutta S. (2020). Emergence of Novel Coronavirus and COVID-19: Whether to Stay or Die Out?. Crit. Rev. Microbiol..

[2] Zhang Y.-Z., Holmes E.C. (2020). A Genomic Perspective on the Origin and Emergence of SARS-CoV-2. Cell.

[3] Zhu Z., Lian X., Su X., Wu W., Marraro G.A., Zeng Y. (2020). From SARS and MERS to COVID-19: A Brief Summary and Comparison of Severe Acute Respiratory Infections Caused by Three Highly Pathogenic Human Coronaviruses. Respir. Res..

[4] Forni D., Cagliani R., Clerici M., Sironi M. (2017). Molecular Evolution of Human Coronavirus Genomes. Trends Microbiol..

[5] Wu A., Peng Y., Huang B., Ding X., Wang X., Niu P., Meng J., Zhu Z., Zhang Z., Wang J. (2020). Genome Composition and Divergence of the Novel Coronavirus (2019-NCoV) Originating in China. Cell Host Microbe.

[6] Gordon D.E., Jang G.M., Bouhaddou M., Xu J., Obernier K., White K.M., O’Meara M.J., Rezelj V.V., Guo J.Z., Swaney D.L. (2020). A SARS-CoV-2 Protein Interaction Map Reveals Targets for Drug Repurposing. Nature.

[7] Huang Y., Yang C., Xu X., Xu W., Liu S. (2020). Structural and Functional Properties of SARS-CoV-2 Spike Protein: Potential Antivirus Drug Development for COVID-19. Acta Pharmacol. Sin..

[8] Sasaki M., Uemura K., Sato A., Toba S., Sanaki T., Maenaka K., Hall W.W., Orba Y., Sawa H. (2021). SARS-CoV-2 Variants with Mutations at the S1/S2 Cleavage Site Are Generated in Vitro during Propagation in TMPRSS2-Deficient Cells. PLoS Pathog..

[9] Shu Y., McCauley J. (2017). GISAID: Global Initiative on Sharing All Influenza Data—From Vision to Reality. Eurosurveillance.

[10] Rambaut A., Holmes E.C., O’Toole Á., Hill V., McCrone J.T., Ruis C., du Plessis L., Pybus O.G. (2020). A Dynamic Nomenclature Proposal for SARS-CoV-2 Lineages to Assist Genomic Epidemiology. Nat. Microbiol..

[11] Magro G. (2020). SARS-CoV-2 and COVID-19: Is Interleukin-6 (IL-6) the ‘Culprit Lesion’ of ARDS Onset? What Is There besides Tocilizumab? SGP130Fc. Cytokine X.

[12] Mokhtari T., Hassani F., Ghaffari N., Ebrahimi B., Yarahmadi A., Hassanzadeh G. (2020). COVID-19 and Multiorgan Failure: A Narrative Review on Potential Mechanisms. J. Mol. Hist..

[13] Wańkowicz P., Szylińska A., Rotter I. (2020). Evaluation of Mental Health Factors among People with Systemic Lupus Erythematosus during the SARS-CoV-2 Pandemic. JCM.

[14] Wańkowicz P., Szylińska A., Rotter I. (2021). The Impact of the COVID-19 Pandemic on Psychological Health and Insomnia among People with Chronic Diseases. JCM.

[15] Andrews M., Areekal B., Rajesh K., Krishnan J., Suryakala R., Krishnan B., Muraly C., Santhosh P. (2020). First Confirmed Case of COVID-19 Infection in India: A Case Report. Indian J. Med. Res..

[16] Seong H., Hyun H.J., Yun J.G., Noh J.Y., Cheong H.J., Kim W.J., Song J.Y. (2021). Comparison of the Second and Third Waves of the COVID-19 Pandemic in South Korea: Importance of Early Public Health Intervention. Int. J. Infect. Dis..

[17] Taboada M., González M., Alvarez A., Eiras M., Costa J., Álvarez J., Seoane-Pillado T. (2021). First, Second and Third Wave of COVID-19. What Have We Changed in the ICU Management of These Patients?. J. Infect..

[18] Gili T., Benelli G., Buscarini E., Canetta C., La Piana G., Merli G., Scartabellati A., Viganò G., Sfogliarini R., Melilli G. (2021). SARS-COV-2 Comorbidity Network and Outcome in Hospitalized Patients in Crema, Italy. PLoS ONE.

[19] Cunningham J.W., Vaduganathan M., Claggett B.L., Jering K.S., Bhatt A.S., Rosenthal N., Solomon S.D. (2021). Clinical Outcomes in Young US Adults Hospitalized With COVID-19. JAMA Intern. Med..

[20] Goldstein E., Lipsitch M., Cevik M. (2021). On the Effect of Age on the Transmission of SARS-CoV-2 in Households, Schools, and the Community. J. Infect. Dis..

[21] Davies N.G., Klepac P., Liu Y., Prem K., Jit M., Eggo R.M., CMMID COVID-19 Working Group (2020). Age-Dependent Effects in the Transmission and Control of COVID-19 Epidemics. Nat. Med..

[22] Jin J.-M., Bai P., He W., Wu F., Liu X.-F., Han D.-M., Liu S., Yang J.-K. (2020). Gender Differences in Patients With COVID-19: Focus on Severity and Mortality. Front. Public Health.

[23] Peckham H., de Gruijter N.M., Raine C., Radziszewska A., Ciurtin C., Wedderburn L.R., Rosser E.C., Webb K., Deakin C.T. (2020). Male Sex Identified by Global COVID-19 Meta-Analysis as a Risk Factor for Death and ITU Admission. Nat. Commun..

[24] Mukherjee S., Pahan K. (2021). Is COVID-19 Gender-Sensitive?. J. Neuroimmune Pharmacol..

[25] Lakbar I., Luque-Paz D., Mege J.-L., Einav S., Leone M. (2020). COVID-19 Gender Susceptibility and Outcomes: A Systematic Review. PLoS ONE.

[26] Elbe S., Buckland-Merrett G. (2017). Data, Disease and Diplomacy: GISAID’s Innovative Contribution to Global Health: Data, Disease and Diplomacy. Glob. Chall..

[27] Thompson R.N., Stockwin J.E., van Gaalen R.D., Polonsky J.A., Kamvar Z.N., Demarsh P.A., Dahlqwist E., Li S., Miguel E., Jombart T. (2019). Improved Inference of Time-Varying Reproduction Numbers during Infectious Disease Outbreaks. Epidemics.

[28] Madewell Z.J., Yang Y., Longini I.M., Halloran M.E., Dean N.E. (2020). Household Transmission of SARS-CoV-2: A Systematic Review and Meta-Analysis. JAMA Netw. Open..

[29] Comunian S., Dongo D., Milani C., Palestini P. (2020). Air Pollution and COVID-19: The Role of Particulate Matter in the Spread and Increase of COVID-19’s Morbidity and Mortality. IJERPH.

[30] Zoran M.A., Savastru R.S., Savastru D.M., Tautan M.N. (2020). Assessing the Relationship between Ground Levels of Ozone (O3) and Nitrogen Dioxide (NO2) with Coronavirus (COVID-19) in Milan, Italy. Sci. Total Environ..

[31] COVID-19 Vaccine Key to Reaching ‘Herd Immunity’. https://www.muhealth.org/our-stories/covid-19-vaccine-key-reaching-herd-immunity.

[32] Young B.E., Wei W.E., Fong S.-W., Mak T.-M., Anderson D.E., Chan Y.-H., Pung R., Heng C.S., Ang L.W., Zheng A.K.E. (2021). Association of SARS-CoV-2 Clades with Clinical, Inflammatory and Virologic Outcomes: An Observational Study. EBioMedicine.

[33] Liu J., Li S., Liu J., Liang B., Wang X., Wang H., Li W., Tong Q., Yi J., Zhao L. (2020). Longitudinal Characteristics of Lymphocyte Responses and Cytokine Profiles in the Peripheral Blood of SARS-CoV-2 Infected Patients. EBioMedicine.

[34] He L., Ding Y., Zhang Q., Che X., He Y., Shen H., Wang H., Li Z., Zhao L., Geng J. (2006). Expression of Elevated Levels of Pro-Inflammatory Cytokines in SARS-CoV-Infected ACE2 ^+^ Cells in SARS Patients: Relation to the Acute Lung Injury and Pathogenesis of SARS. J. Pathol..

[35] Esper F.P., Cheng Y.-W., Adhikari T.M., Tu Z.J., Li D., Li E.A., Farkas D.H., Procop G.W., Ko J.S., Chan T.A. (2021). Genomic Epidemiology of SARS-CoV-2 Infection During the Initial Pandemic Wave and Association With Disease Severity. JAMA Netw. Open.

[36] Pandit B., Bhattacharjee S., Bhattacharjee B. (2021). Association of Clade-G SARS-CoV-2 Viruses and Age with Increased Mortality Rates across 57 Countries and India. Infect. Genet. Evol..

[37] Nakamichi K., Shen J.Z., Lee C.S., Lee A., Roberts E.A., Simonson P.D., Roychoudhury P., Andriesen J., Randhawa A.K., Mathias P.C. (2021). Hospitalization and Mortality Associated with SARS-CoV-2 Viral Clades in COVID-19. Sci. Rep..

[38] Hamed S.M., Elkhatib W.F., Khairalla A.S., Noreddin A.M. (2021). Global Dynamics of SARS-CoV-2 Clades and Their Relation to COVID-19 Epidemiology. Sci. Rep..

[39] Takahashi T., Ellingson M.K., Wong P., Israelow B., Lucas C., Klein J., Silva J., Mao T., Oh J.E., Yale IMPACT Research Team (2020). Sex Differences in Immune Responses That Underlie COVID-19 Disease Outcomes. Nature.

[40] Marimuthu S., Joy M., Malavika B., Nadaraj A., Asirvatham E.S., Jeyaseelan L. (2021). Modelling of Reproduction Number for COVID-19 in India and High Incidence States. Clin. Epidemiol. Glob. Health.

[41] Ranjan R., Sharma A., Verma M.K. (2021). Characterization of the Second Wave of COVID-19 in India. medRxiv.

